# The Physician

**Published:** 1856-01

**Authors:** 


*The Physician.*—Here is a tribute well deserved, to a profession to which society owes a vast debt:

"No class of men in the regular discharge of duty, incur danger more frequently than the honest physician. There is no type of malignant maladies with which he fails to become acquainted; no hospital so crowded with contagion that he dares not walk freely through its wards. His vocation is among the sick and dying; he is the familiar friend of those who are sinking under infectious disease; and he never shrinks from the horror of observing it under all its aspects. He must do so with equanimity; as he inhales the poisoned atmosphere, he must coolly reflect on the medicines which may mitigate the sufferings that he cannot remedy. Nay, after death has ensued, he must search with the dissecting knife for its hidden cause, if so by multiplying his own perils he may discover some alleviation for the afflictions of others. And why is this? Because the physician is indifferent to death? Because he is steeled and hardened against the fear of it? Because he despises or pretends to despise it? By no means. It is his especial business to value life; to cherish the least spark of animated existence. And the habit of caring for the lives of his fellow-men is far from leading him to an habitual indifference to his own. The physician shuns every danger but such as the glory of his profession commands him to defy."—*Boston Med. & Surg. Journ.*

